# Comprehensive Lower Extremities Joints Range of Motion Profile in Futsal Players

**DOI:** 10.3389/fpsyg.2021.658996

**Published:** 2021-06-14

**Authors:** Antonio Cejudo, Iñaki Ruiz-Pérez, Sergio Hernández-Sánchez, Mark De Ste Croix, Pilar Sainz de Baranda, Francisco Ayala

**Affiliations:** ^1^Department of Physical Activity and Sport, Faculty of Sports Sciences, University of Murcia, Murcia, Spain; ^2^Department of Sport Sciences, Sports Research Centre, Miguel Hernández University of Elche, Elche, Spain; ^3^Department of Pathology and Surgery, Physiotherapy Area, Miguel Hernandez University of Elche, Alicante, Spain; ^4^School of Sport and Exercise, University of Gloucestershire, Gloucester, United Kingdom; ^5^Ramón y Cajal Postdoctoral Fellowship, Department of Physical Activity and Sport, Faculty of Sports Sciences, University of Murcia, Murcia, Spain

**Keywords:** futsal, muscle flexibility, injury risk, soft tissue injuries, rehabilitation

## Abstract

The purposes of this study were to describe the lower extremities joints range of motion (ROM) profile using a comprehensive approach in futsal players and to examine potential player position (goalkeepers vs. outfield players), competitive level (first [top] division vs. second division), number of playing years, sex (males vs. females), and bilateral (dominant limb vs. non-dominant limb) differences. A total of 72 male and 67 female elite futsal players from 11 clubs were measured of passive hip (flexion with knee flexed [HF_*KF*_] and extended [HF_*KE*_], extension [HE], abduction [HA], external [HER], and internal [HIR] rotation), knee (flexion [KF]) and ankle (dorsiflexion with knee flexed [ADF_*KF*_] and extended [ADF_*KE*_]) ROMs. Bayesian inferences exploring differences between player position, competitive level, sex and limb were made. A Bayesian correlation analysis was conducted to explore the influence of playing years on joints ROMs. The results showed no significant player position or competitive level related differences in any average ROM score. However, statistically significant sex-related differences were documented whereby female players reported higher hip and knee joints ROM average values than their male counterparts. Especially relevant were the proportions of males (72%) and players from teams engaged in the second division (61%) displaying limited HF_*KE*_ ROMs. Likewise, around 35% of all players showed restricted ADF_*KF*_ ROMs. In addition, approximately 21, 18, 22, and 25% of the futsal players were identified as having bilateral asymmetries (≥8°) for HA, HIR, HER, and KF ROMs, respectively. Finally, Bayesian correlation analysis did not report any significant association between years of playing futsal and ROM measures (all *r* values < 0.34). The implications that these restricted HF_*KE*_ and ADF_*KF*_ ROMs and bilateral asymmetries in hip (abduction, internal and external rotation) and knee (flexion) ROMs caused by the practice of futsal may have on physical performance and injury risk warrant future research.

## Introduction

Futsal (the five-a-side version of associated football) is played worldwide with more than one million registered players all over the world (FIFA, 2007). During the game of futsal, players perform a substantive number of repeated high intensity unilateral actions (e.g., sudden accelerations and decelerations, rapid changes of direction, tackling and kicking) (Dogramaci and Watsford, 2006; Castagna et al., 2009; Beato et al., 2016; Naser et al., 2017). Such high intensity actions alongside lateral preference (e.g., preferred kicking leg) might lead players to progressively develop futsal-specific soft tissue adaptations (Maloney, 2019). In particular, most of these actions impose strong and asymmetric tensile loads on the muscles around the hip, knee, and ankle joints. When these actions are repeated several times during training sessions and matches, they may have the potential to generate changes in the mechanical (e.g., stiffness) and neural (e.g., tolerance to changes in resting length) properties of one or some of the muscle groups (mainly biarticular) involved in them (Witvrouw et al., 2004). Thus, it may be plausible that these muscle adaptations are likely to result in the development of a futsal-specific lower extremities joints range of motion (ROM) profile that might be characterized by the presence of some restricted or limited ROMs and significant bilateral asymmetries (dominant limb vs. non-dominant limb). Furthermore, this hypothetical futsal-specific lower extremities ROM profile may become more evident at elite levels, mainly attributed (but not exclusively) to the higher physical demands of the game and larger number of training sessions per week that players at these levels are usually exposed to Mohammed et al. (2014), Ribeiro et al. (2020), Spyrou et al. (2020). Likewise, the well-documented sex-related anatomical (Hahn and Foldspang, 1997), hormonal (Wojtys et al., 1998), and neuromuscular (Komi and Karlsson, 1978) differences may lead female futsal players to develop a different lower extremities ROM profile in comparison with their male counterparts.

It has been traditionally suggested that studies aimed at describing the effects of long-standing participation within a single sport on major joints ROMs are of interest because restricted values and bilateral asymmetries are not only detrimental to physical performance but also increase the risk of soft tissue injury (Bishop et al., 2018; Afonso et al., 2020). However, from a physical performance standpoint the available evidence does not support such association. In this sense, the findings reported by the studies that have addressed this issue in intermittent team-sport athletes (mainly football and basketball players) are often contradictory, whereby for the same physical performance measure (e.g., jump height), some studies exhibited negative associations (García-Pinillos et al., 2015; Mills et al., 2015) while others did not find a clear influence (Domínguez-Díez et al., 2021) and even better scores were observed in players with poor ROM values (Rey et al., 2016). On the other hand, a growing number of prospective studies have been recently published using contemporary Machine Learning techniques (e.g., supervised learning algorithms) and resampling methods (e.g., fivefold cross validation, leave-one-out, bootstrapping) to build valid and generalizable screening models (area under the receiver operator characteristics [ROC] scores > 0.700) to predict non-contact soft-tissue lower extremities injuries in intermittent team-sport athletes (including futsal players) (Fousekis et al., 2011; López-Valenciano et al., 2018; Rossi et al., 2018; Ayala et al., 2019; Oliver et al., 2020; Rommers et al., 2020; Ruiz-Pérez et al., 2021). Among these studies, those that provided learning algorithms the opportunity to select (or not) measures of ROMs to build prediction models have identified some restricted lower extremities hip (flexion), knee (flexion), and ankle (dorsiflexion) ROMs and bilateral asymmetries as primary predictors of non-contact soft-tissue injury (mainly thigh muscle strains and knee and ankle ligament sprains and tears) in football (López-Valenciano et al., 2018; Ayala et al., 2019), handball (López-Valenciano et al., 2018), and futsal players (Ruiz-Pérez et al., 2021). Therefore, and with a certain degree of caution, it could be stated that knowing whether (or not) futsal players may develop sport-specific adaptations from training and matches that would cause significant impairments and bilateral differences in lower extremities joints ROMs might help coaches and sport scientists in the decision-making process for injury prevention.

Only Cejudo et al. (2014) have described the lower extremities ROM profile in elite male futsal players. The results shown in this study indicate that the practice of futsal did not elicit clinically relevant impairments in hip, knee and ankle joints ROM average values in this cohort of players. Likewise, no statistically significant bilateral differences were found for the joint ROMs of the dominant and non-dominant limbs. However, the lower extremities ROM profile described by Cejudo et al. (2014) should be considered with a degree of caution since only 20 players from a single futsal team were used, which may reduce its external validity. In addition, Cejudo et al. (2014) only provided average values so that the possible inter-player variability in joint ROMs was not considered and thus, it may distort the true extent of the number of players reporting restricted ROMs. In an attempt to minimize the effects of inter-player variability and achieve a more realistic diagnosis regarding the presence (or absence) of changes in ROMs attributed to intensive sport practice, López-Valenciano et al. (2019) suggested using a new comprehensive profile of joint ROMs. In this profile not only ROM average scores are reported but also the number of players showing bilateral asymmetries (between-limb differences > 6–10°) (Fousekis et al., 2011; López-Valenciano et al., 2019) and normal and limited (based on the previously published cutoff scores to classify athletes at high risk of injury) ROM values.

Therefore, the purposes of this study were to describe the lower extremities joints ROM profile using a comprehensive approach in a large cohort of futsal players and to examine potential player position (goalkeepers vs. outfield players), competitive level (first [top] division vs. second division), number of playing years, sex (males vs. females), and bilateral (dominant limb vs. non-dominant limb) differences.

## Materials and Methods

### Participants

A convenience sample of 139 (72 males and 67 females) elite futsal players from 12 different teams (56 players [24 males and 32 females] from six clubs engaged in the First [top] National Spanish Futsal division and 83 players [48 males and 35 females] from six clubs engaged in the Second National Futsal division) completed this study. Before data collection, participants filled out a questionnaire containing questions about their sport-related background (player position, the current competitive level, dominant leg, sport experience), anthropometric characteristics (age, body mass, and stature) and training regimen (weekly practice frequency, hours of futsal practice per week and day, number of playing years). Descriptive statistics for males and females are displayed in Table 1. The exclusion criterion was history of orthopedic problems to the knee, thigh, hip, or lower back in the month before the study from which players were considered (by teams’ medical staff) as not fully recovered and whose acute residual symptoms could have a temporary impact on the habitual players’ movement competency and/or lower extremities ROM profile (López-Valenciano et al., 2019; Moreno-Pérez et al., 2020). The study was conducted at the end of the pre-season phase in 2015 (39 male [4 teams] players), 2016 (26 male [2 teams] and 18 female [2 teams] players), 2017 (7 male [1 team] and 23 female [2 teams] players) and 2018 (26 female [2 teams] players) (September). Only one pre-season ROM assessment was carried out in 121 out of 139 players through the 4-year length of the study while 18 female players from the same team were assessed twice in different years (2017 and 2018). The time frame of the study was selected to be sure that the players recruited to each team were definitive and stable within the testing period. Before any participation, experimental procedures and potential risks were fully explained to the players and coaches in verbal and written form and written informed consent was obtained from players. An Institutional Research Ethics committee approved the study protocol prior to data collection (DPS.FAR.01.14) conforming to the recommendations of the Declaration of Frontera.

**TABLE 1:** Demographic variables (mean ± SD) for the elite futsal players.

Variable	Males (*n* = 72)	Females (*n* = 67)
Age (years)	22.8 ± 5.5	22.3 ± 4.5
Stature (cm)	177.6 ± 6.4	164.6 ± 5.8
Body mass (kg)	73.1 ± 6.6	60.2 ± 6.7
Years playing futsal (years)	13.8 ± 5.1	13.2 ± 4.4
Weekly practice frequency	4.1 ± 0.6	3.1 ± 0.4
Hours of futsal practice per week	10.8 ± 1.7	8.4 ± 1.0

*SD, standard deviation.*

### Testing Procedure

The passive hip flexion with knee flexed (HF_*KF*_) and extended (HF_*KE*_), extension (HE), abduction (HA), external (HER) and internal (HIR) rotation; knee flexion (KF); and ankle dorsiflexion with knee flexed (ADF_*KF*_) and extended (ADF_*KE*_) ROMs of the dominant and non-dominant limbs were assessed following the methodology previously described (Cejudo et al., 2020). Briefly, an ISOMED inclinometer (Portland, Oregon) with a telescopic arm was used as the key measure for all tests. The inclinometer was consistently placed level before each measurement to assure that no change occurred in the sensitivity. A low-back protection sup- port (Lumbosant, Murcia, Spain) was used to standardize the lordotic curve (15°) during all the assessment tests. Variations in pelvic position and stability may affect the final score of several measurements of hip movement range of motion (Bohannon et al., 1985). Thus, to accurately measure hip joint ROMs, the assessment procedure in this study provided suitable stabilization of the pelvis during all the tests using an assistant clinician.

The dominant limb was defined as the participant’s preferred kicking leg (self-reported). Prior to the testing session, all participants performed the dynamic warm-up designed by Taylor et al. (2009). The overall duration of the entire warm-up was approximately 20 min. The assessment of the nine ROMs was carried out 3–5 min after the dynamic warm-up. After the warm-up, participants were instructed to perform, in a randomized order, two maximal trials of each ROM test for each limb, and the mean score for each test was used in the analyses. When a variation > 4° was found in the ROM values between the two trials of any test, an extra trial was performed, and the two most closely related trials were used for the subsequent statistical analyses. One or both of the following criteria determined the endpoint for each test: (a) palpable onset of pelvic rotation, and/or (b) the participant feeling a strong but tolerable stretch, slightly before the occurrence of pain. Participants were examined wearing sports clothes and without shoes. A 30 s rest was given between trials, limbs and tests. All tests were carried out by the same two sports science specialists under stable environmental conditions. Standardization procedures (including the warm-up, test setup, and participant instructions) were replicated at each test session conducted in the different clubs. Teams’ performance staff were told not to request players to perform high intensity activities at least 48 h before testing sessions to minimize the impact of potential muscle soreness and contractures on ROM scores. All testing sessions were carried out within the 2 weeks before the beginning of the futsal in-season and always in the late afternoon or evening (according to each team’ training schedules).

### Statistical Analysis

Statistical analyses were performed using JASP software version 0.13.01 (Amsterdam, Netherland). Prior to the statistical analysis, the distribution of raw data sets was checked using the Shapiro–Wilk expanded test and demonstrated that all data had a normal distribution (*p* > 0.05). Descriptive statistics (including means and standard deviations [SD]) were calculated for hip, knee and ankle ROM measures for all futsal players combined and also separately by player position (goalkeepers vs. outfield players), competitive level (first division vs. second division) and sex (males vs. females).

Separate Bayesian paired samples *t*-tests were carried out to determine the existence of significant bilateral differences in hip, knee and ankle average ROMs. Likewise, Bayesian independent samples *t*-tests were conducted to explore differences in ROM average scores between player position, competitive level and sex. For all the Bayesian inference tests run, the BF_10_ was interpreted using the evidence categories previously suggested (Wagenmakers et al., 2018): < $\frac{1}{100}$ = extreme evidence for null hypothesis (H_0_ = no main effects), from $\frac{1}{100}$ to <$\frac{1}{30}$ = very strong evidence for H_0_, from $\frac{1}{30}$ to <$\frac{1}{10}$ = strong evidence for H_0_, from $\frac{1}{10}$ to <$\frac{1}{3}$ = moderate evidence for H_0_, from $\frac{1}{3}$ to < 1 anecdotical evidence for H_0_, from 1 to 3 = anecdotical evidence for alternative hypothesis (H_1_), from >3–10 = moderate evidence for H_1_, from >10 to 30 = strong evidence for H_1_, from >30–100 = very strong evidence for H_1_, > 100 extreme evidence for H_1_. Only those models that showed at least strong evidence for supporting H_1_ (BF_10_ > 10) with a percental error < 10 were considered robust enough to describe the main effects.

Furthermore, and similar to what was carried out in previous studies on football players (Ayala et al., 2019; Robles-Palazón et al., 2020), in each participant, the hip, knee and ankle ROM scores were categorized as normal (i.e., non-pathologic) or limited according to the reference values previously reported to consider an athlete as being more prone to suffer an injury. Thus, ROM values were reported as limited according to the following cut-off scores: <114° (limited) and ≥114° (normal) for the HF_*KF*_ ROM (Holla et al., 2012), <80° (limited) and ≥80° (normal) for the HF_*KE*_ ROM (Kendall et al., 2005), <50° (limited) and ≥50° (normal) for the HA ROM (Gerhardt et al., 2002), <26° (limited) and ≥26° (normal) for the HIR ROM (Roach et al., 2013), <30° (limited) and ≥30° (normal) for the HER ROM (L’Hermette et al., 2006), <0° (limited) and ≥0° (normal) for the HE ROM (Young et al., 2004), <120° (limited) and ≥120° (normal) for the KF ROM (Peat et al., 2007), <17° (limited) and ≥17° (normal) for the ADF_*KE*_ ROM (Ekstrand and Gillquist, 1982; Kibler et al., 1988) and <34° (limited) and ≥34° (normal) for the ADF_*KF*_ ROM (Pope et al., 1998). In those ROMs in which different cut-off scores have been described in the literature to categorize athletes as having either high or moderate risk of soft-tissue injury, the most restrictive one was selected as the cut-off score for the current study. The cut-off scores previously suggested by Robles-Palazón et al. (2020) were used to calculate the number and percentage of players with bilateral differences (≥8°) in each ROM. For each ROM, percentage scores larger than 20% of players showing limited values and significant bilateral differences were considered for this study as relevant from an injury prevention standpoint. In each ROM in which the percentage of players showing limited values and/or significant bilateral differences were larger than 20%, a Bayesian Pearson’s chi-squared (*x*^2^) test was used to examine potential player position (goalkeepers vs. outfield players), competitive level (first [top] division vs. second division) and sex (males vs. females) -related differences in the proportion of players showing limited scores and bilateral differences.

Finally, a Bayesian correlation analysis was performed to examine the correlation between participants’ years of playing futsal and each ROM score. Magnitudes of correlations were assessed using the following scale of thresholds: <0.80 = low, 0.80–0.90 = moderate, and >0.90 = high (Hopkins, 2000).

## Results

Tables 2–5 display the descriptive ROM values for hip (HF_*KF*_, HF_*KE*_, HA, HIR, HER and HE), knee (KF), and ankle (ADF_*KF*_ and ADF_*KE*_) joints for all players combined and separately by player position, competitive level and sex, respectively.

**TABLE 2:** Descriptive values and inter-limb differences for hip (flexion, extension, abduction, internal, and external rotation), knee (flexion), and ankle (dorsalflexion with knee flexed and extended) ranges of motion for all futsal players combined (*n* = 139).

Range of motion (°)	Dominant limb		Non-dominant limb		Inter-limb differences	
	Mean ± SD	Qualitative outcome^a^	Mean ± SD	Qualitative outcome^a^	Mean and 95% CI	Players with bilateral differences ≥ 8°
HF_*KF*_	136.1 ± 9.5	Normal (1/139)	137.4 ± 9.3	Normal (1/139)	–1.3 (–2.1 to –0.4)	21/139 (15%)
HF_*KE*_	83.2 ± 16	Normal (66/139)	82.9 ± 15.3	Normal (62/139)	0.3 (–0.6 to 1.2)	24/139 (17%)
HA	64.4 ± 11.8	Normal (12/139)	63.4 ± 12.6	Normal (12/139)	1 (–0.1 to 1.9)	29/139 (21%)
HIR	47.6 ± 10.4	Normal (2/139)	46.7 ± 10.9	Normal (1/139)	0.9 (–0.3 to 2)	39/139 (28%)
HER	57.8 ± 9.2	Normal (0/139)	57.2 ± 10.1	Normal (0/139)	0.7 (–0.5 to 1.8)	30/139 (22%)
HE	14.6 ± 7.5	Normal (3/139)	15.1 ± 7.5	Normal (3/139)	–0.5 (–0.9 to –0.1)	4/139 (3%)
KF	128.4 ± 11.3	Normal (0/139)	126.3 ± 10.6	Normal (0/139)	2.1 (0.9 to 3.2)	35/139 (25%)
ADF_*KE*_	33.9 ± 5.4	Normal (1/139)	33.2 ± 4.8	Normal (0/139)	0.6 (0 to 1.2)	10/139 (7%)
ADF_*KF*_	35.8 ± 5.6	Normal (49/139)	36 ± 5.8	Normal (45/139)	–0.2 (–0.7 to 0.4)	7/139 (5%)

*HF_KF_, hip flexion with knee flexed test; HF_KE_, hip flexion with knee extended test; HA, hip abduction test; HIR, hip internal rotation test; HER, hip external rotation test; HE, hip extension test; KF, knee flexion test; ADF_KE_, ankle dorsiflexion with knee extended test; ADF_KF_, ankle dorsiflexion with knee flexed test. °, degrees; ^a^, qualitative score of the mean range of motion, in parentheses the number of players with a limited range of motion score according to previously published cut-off scores (see section “Statistical analysis”).*

**TABLE 3:** Descriptive values and number (percentage) of players with bilateral differences ≥ 8° for hip (flexion, extension, abduction, internal and external rotation), knee (flexion), and ankle (dorsal-flexion with knee flexed and extended) ranges of motion separately by player position.

Range of motion (°)	Goalkeepers (*n* = 31)			Outfield players (*n* = 108)		
	Mean ± SD	Qualitative outcome^a^	Players with bilateral differences ≥ 8°	Mean ± SD	Qualitative outcome^a^	Players with bilateral differences ≥ 8°
HF_*KF*_	138.1 ± 10.2	Normal (0/31)	3/31 (10%)	136.1 ± 9.8	Normal (1/108)	18/108 (17%)
HF_*KE*_	87.5 ± 16.2	Normal (13/31)	6/31 (19%)	83.2 ± 16.5	Normal (53/108)	18/108 (17%)
HA	69.7 ± 10.9	Normal (0/31)	5/31 (16%)	63.6 ± 12.3	Normal (12/108)	24/108 (22%)
HIR	48.6 ± 8.8	Normal (0/31)	7/31 (23%)	47.9 ± 11.2	Normal (2/108)	32/108 (30%)
HER	61.1 ± 8.7	Normal (0/31)	7/31 (23%)	57.5 ± 9.7	Normal (0/108)	23/108 (21%)
HE	16.4 ± 8.2	Normal (1/31)	2/31 (6%)	14.5 ± 7.6	Normal (2/108)	2/108 (2%)
KF	131 ± 8.7	Normal (4/31)	8/31 (26%)	128.1 ± 12.4	Normal (22/108)	27/108 (25%)
ADF_*KE*_	33 ± 6.8	Normal (0/31)	3/31 (10%)	34.1 ± 5.2	Normal (0/108)	7/108 (7%)
ADF_*KF*_	34.2 ± 6.2	Normal (13/31)	0/31 (0%)	36.1 ± 5.6	Normal (36/108)	7/108 (7%)

*HF_KF_, hip flexion with knee flexed test; HF_KE_, hip flexion with knee extended test; HA, hip abduction test; HIR, hip internal rotation test; HER, hip external rotation test; HE, hip extension test; KF, knee flexion test; ADF_KE_, ankle dorsiflexion with knee extended test; ADF_KF_, Ankle dorsiflexion with knee flexed test. °, degrees; ^a^, qualitative score of the mean range of motion, in parentheses the number of players with a limited range of motion score according to previously published cut-off scores (see section “Statistical analysis”).*

**TABLE 4:** Descriptive values and number (percentage) of players with bilateral differences ≥ 8° for hip (flexion, extension, abduction, internal and external rotation), knee (flexion), and ankle (dorsal-flexion with knee flexed and extended) ranges of motion separately by competitive level.

Range of motion (°)	First division (*n* = 56)			Second division (*n* = 83)		
	Mean ± SD	Qualitative outcome^a^	Players with bilateral differences ≥ 8°	Mean ± SD	Qualitative outcome^a^	Players with bilateral differences ≥ 8°
HF_*KF*_	138.2 ± 10	Normal (0/56)	11/56 (20%)	134.7 ± 8.9	Normal (0/83)	10/83 (12%)
HF_*KE*_	86.1 ± 14.4	Normal (15/56)	13/56 (23%)	81.2 ± 16.8	Normal (51/83)	11/83 (13%)
HA	64.9 ± 11.8	Normal (6/56)	16/56 (29%)	64.1 ± 11.9	Normal (6/83)	13/83 (16%)
HIR	48.5 ± 9.9	Normal (1/56)	14/56 (25%)	46.9 ± 10.7	Normal (1/83)	25/83 (30%)
HER	59.9 ± 10.1	Normal (0/56)	13/56 (23%)	56.4 ± 8.4	Normal (0/83)	17/83 (21%)
HE	12.6 ± 6.6	Normal (1/56)	3/56 (5%)	15.9 ± 7.8	Normal (2/83)	1/83 (1%)
KF	125.3 ± 9.2	Normal (13/56)	11/56 (20%)	130.4 ± 12.1	Normal (13/83)	24/83 (29%)
ADF_*KE*_	34 ± 5.5	Normal (1/56)	4/56 (7%)	33.8 ± 5.4	Normal (0/83)	6/83 (7%)
ADF_*KF*_	35.2 ± 4.8	Normal (22/56)	4/56 (7%)	36.3 ± 6	Normal (27/83)	3/83 (4%)

*HF_KF_, hip flexion with knee flexed test; HF_KE_, hip flexion with knee extended test; HA, hip abduction test; HIR, hip internal rotation test; HER, hip external rotation test; HE, hip extension test; KF, knee flexion test; ADF_KE_, ankle dorsiflexion with knee extended test; ADF_KF_, ankle dorsiflexion with knee flexed test. °, degrees; ^a,^ qualitative score of the mean range of motion, in parentheses the number of players with a limited range of motion score according to previously published cut-off scores (see section “Statistical analysis”).*

**TABLE 5:** Descriptive values and number (percentage) of players with bilateral differences ≥ 8° for hip (flexion, extension, abduction, internal and external rotation), knee (flexion), and ankle (dorsal-flexion with knee flexed and extended) ranges of motion separately by sex.

Range of motion (°)	Males (*n* = 72)			Females (*n* = 67)		
	Mean ± SD	Qualitative outcome^a^	Players with bilateral differences ≥ 8°	Mean ± SD	Qualitative outcome^a^	Players with bilateral differences ≥ 8°
HF_KF_*	132.3 ± 9.2	Normal (1/72)	6/72 (8%)	140.2 ± 8.2	Normal (0/67)	15/67 (22%)
HF_KE_*	74.1 ± 9.7	Limited (52/72)	10/72 (14%)	92.9 ± 15.8	Normal (14/67)	14/67 (21%)
HA*	58.4 ± 7.6	Normal (10/72)	13/72 (18%)	70.9 ± 12.1	Normal (2/67)	16/67 (24%)
HIR*	41.8 ± 6.8	Normal (1/72)	13/72 (18%)	53.8 ± 10.0	Normal (1/67)	26/67 (39%)
HER*	54 ± 7.6	Normal (0/72)	17/72 (24%)	61.9 ± 9.2	Normal (0/67)	13/67 (19%)
HE*	11.9 ± 6.3	Normal (2/72)	0/72 (0%)	17.6 ± 7.5	Normal (1/67)	4/67 (6%)
KF*	125.4 ± 9.7	Normal (17/72)	13/72 (18%)	131.6 ± 12.1	Normal (9/67)	22/67 (33%)
ADF_*KE*_	33.4 ± 4.5	Normal (0/72)	3/72 (4%)	34.3 ± 6.2	Normal (1/67)	7/67 (10%)
ADF_*KF*_	35.7 ± 5.6	Normal (25/72)	3/72 (4%)	36 ± 5.6	Normal (24/67)	4/67 (6%)

HF_KF_, hip flexion with knee flexed test; HF_KE_, hip flexion with knee extended test; HA, hip abduction test; HIR, hip internal rotation test; HER, hip external rotation test; HE, hip extension test; KF, knee flexion test; ADF_KE_, ankle dorsiflexion with knee extended test; ADF_KF_, ankle dorsiflexion with knee flexed test. °, degrees; ^a^, qualitative score of the mean range of motion, in parentheses the number of players with a limited range of motion score according to previously published cut-off scores (see section “Statistical analysis”). *, statistically significant differences (BF_10_ > 10) between sex (males vs. females).

With all players combined, Bayesian paired samples *t*-tests did show no significant differences (BF_10_ < 10) between dominant and non-dominant limbs for each of the nine ROMs (Table 2). Consequently, the ROM average score for both limbs was used for between-group comparisons. There were no significant differences in the hip, knee and ankle joints ROM values obtained from goalkeepers and outfield players (Table 3). Likewise, there were also no significant competitive level related differences in any of the ROMs (Table 4). However, statistically significant sex-related differences were documented whereby female players reported higher hip and knee joints ROM average values than their male counterparts (Table 5).

The comprehensive analysis conducted in this study found that a significant number of the futsal players (independently of the player position, competitive level and sex) demonstrate limited HF_*KE*_ (≈47%) and/or ADF_*KF*_ (35%) ROMs (Table 2). In addition, approximately 21, 18, 22, and 25% of the futsal players were identified as having bilateral asymmetries (≥8°) for HA, HIR, HER, and KF ROMs, respectively. Inter-group comparisons showed that percentages larger than 40% of the goalkeepers and outfield players recruited in this study presented limited HF_*KE*_ and ADF_*KF*_ ROMs. Likewise, significant bilateral differences in HIR and HER ROMs were observed for 23% of the goalkeepers and 23% (HIR ROM) and 30% (HER ROM) of the outfield players. The Bayesian *x*^2^ tests did not show significant differences between the proportions of goalkeepers and outfield players with limited HF_*KE*_ and ADF_*KF*_ ROMs nor between those goalkeepers and outfield players who displayed bilateral differences ≥ 8° in these two ROMs. Approximately 26, 23, and 39% of players from teams engaged in the first division reported limited HF_*KE*_, KF and ADF_*KF*_ ROM values, respectively. Furthermore, percentages of first division players ranged from 23 to 29% presented significant bilateral differences in their HF_*KE*_, HA, HIR, and HER ROMs. For its part, a percentage larger than 20% of the players from teams engaged in the second division showed limited HF_*KE*_ (77%) and ADF_*KF*_ (32%) ROMs as well as bilateral differences ≥ 8° in their HIR (30%), HER (31%), and KF (29%) ROMs. Significant differences in the proportion of first and second division players with limited ROM values were only found for the HF_*KE*_ ROM (23% [first division] vs. 77% [second division]) (BF_10 *Poisson*_ > 10). However, no significant differences were found between the proportions of first division and second division players who displayed bilateral differences ≥ 8° in their ROMs. Approximately 72, 24, and 35% of the male players displayed restrictions in their HF_*KE*_, KF and ADF_*KF*_ ROM values, respectively. For females, 21 and 36% of them reported limited HF_*KE*_ and ADF_*KF*_ ROM values. The proportion of female players with limited HF_*KE*_ ROM values was significantly lower than that of males (BF_10 *Poisson*_ > 10). Likewise, 24% of the male players showed significant bilateral differences in their HER ROM whereas 22, 21, 24, 39, and 32% of the females presented inter-limb differences ≥ 8° in their HF_*KF*_, HF_*KE*_, HA, HIR and KF ROMs. The proportion of female players with bilateral differences ≥ 8° in the HF_*KE*_ and HIR was significantly higher than that of males (18% [males] vs. 39% [females] and 18% [males] vs. 32% [females] for HIR and HF_*KE*_, respectively).

Finally, Bayesian correlation analysis did not report any significant correlation between years of playing futsal and ROM measures (all *r* values < 0.34).

## Discussion

The findings of this study indicate that the average values of the nine ROMs assessed in the futsal players may be categorized as normal or non-limited according to the cutoff scores described in the literature to identify athletes at high risk of sustaining a soft-tissue injury. Similar results were found by Cejudo et al. (2014), who after having carried out the same ROM maneuvers and testing procedures [ROM-Sport protocol (Cejudo et al., 2020)] in male futsal players, found hip, knee and ankle ROM average values that may be categorized as normal. From this standpoint, no specific adaptations in the lower extremities joints ROM would be expected as a consequence of futsal training and match play at elite levels. However, when a comprehensive analysis is carried out, the current ROM data shows that a significant number of the futsal players demonstrate limited HF_*KE*_ (≈47%) and/or ADF_*KF*_ (≈35%) ROMs, irrespective of their position, competitive level and sex. Previous studies using the same comprehensive analysis employed in the current study also identified a large number of youth (Robles-Palazón et al., 2020) and adult (López-Valenciano et al., 2019) male football players with limited HF_*KE*_ (≈45%) and/or ADF_*KF*_ (30%) ROMs, despite the fact that their pooled average scores for these two ROMs were categorized as normal or non-limited [HF_*KE*_ > 80° (Kendall et al., 2005) and ADF_*KF*_ > 34° (Pope et al., 1998)]. Therefore, collectively these findings support the statement that an accurate diagnosis of the sport-specific adaptations in the lower extremities joints ROM requires reporting not only ROM average scores but also the number of athletes showing limited (based on the previously published cutoff scores to classify athletes at high risk of injury) ROMs. Similar to that which has been argued for football players, a plausible explanation for the large percentage of futsal players demonstrating limited HF_*KE*_ (≈47%) and/or ADF_*KF*_ (35%) ROMs may be based on the demands of the game of futsal that requires players to perform several repeated high intensity movements such as sudden acceleration and deceleration, rapid changes of directions, kicking and tackling (Naser et al., 2017). These movements impose strong concentric and eccentric loads on the hip flexors and ankle dorsiflexion muscles (posterior kinetic chain) at shortened contracted positions (Orchard, 2012; Sun et al., 2015). When these actions are repeated several times during training sessions and matches, they have the potential to generate muscle damage that without the proper recovery and protective measures, might induce impairments in the mechanical and neural properties of the muscle-tendon units, including a reduction in their normal ROMs (Fridén and Lieber, 2001).

The findings of this study also show that, and unlike the between-subject factors player position and competitive level, there was a significant main effect (BF_10_ > 10 [at least a strong evidence in favor of H_1_]) for the factor sex on the ROM profile of the futsal players tested whereby females presented higher hip and knee ROM average values than male players. However, it should be noted that for both males and females, all ROM average values (except for the HF_*KE*_ ROM values [74.1 ± 9.7°] documented for the males that were cataloged as limited [<80°]) were categorized as normal or non-limited. On the other hand, the comprehensive analysis conducted in this study also revealed that more than 20% of male and female players displayed restrictions in their HF_*KE*_ and ADF_*KF*_ ROM values. When potential sex-related differences in the proportion of players displaying limited HF_*KE*_ and ADF_*KF*_ ROM values were explored, only statistically significant differences were observed in favor of the males for the HF_*KE*_ ROM (72% [males] vs. 21% [females]). In practical terms, approximately 3 out of 4 diagnosed cases of limited HF_*KE*_ ROM found in futsal players may be expected to be males. The fact that females presented lower volume of futsal practice per week than males (8.4 h vs. 10.8 h) alongside the larger number of high intensity movements that males perform during matches in comparison with females (Beato et al., 2017; Naser et al., 2017; Serrano et al., 2020) may have contributed to these sex-related differences on the lower extremities ROM profile of the futsal players. Furthermore, unlike the monoarticular ankle dorsiflexion muscles (i.e., soleus and gastrocnemius), the biarticular nature of the hip flexor muscles (i.e., hamstrings) foster them to be repeatedly subjected to the high loading forces generated during most of the futsal-specific movements and this together with the just-mentioned higher exposure to futsal play observed in males as opposed to female players may explain the sex-related differences in the proportion of players with limited HF_*KE*_ and the absence of them in the ADF_*KF*_ ROM. Although the current evidence does not allow to make strong claims with regard to the potential effects that limited ROMs may elicit on injury risk, the significant proportion of male futsal players exhibiting limited HF_*KE*_ ROMs (72%) may help elucidate the reasons why they show a high predisposition to suffer hip flexor muscles strains. For this specific HF_*KE*_ ROM, the results also report that futsal teams engaged in the first division presented a lower proportion of players with limited values than their counterparts playing in the second division (27% vs. 61%). It is likely that the differences in terms of professionalism (e.g., number of medical and performance staff members, available testing, and training equipment), match physical demands and training status of players between futsal’s first and second divisions may justify why the proportion of players showing limited HF_*KE*_ ROM was lower in teams engaged in the first division than in the second division.

Despite having been considered as an asymmetrical sport (Barbieri et al., 2015), the results of the current study along with the findings of Cejudo et al. (2014), describe non-significant bilateral differences (≥8°) between the dominant and non-dominant lower extremities joints ROM average values in futsal players (independent of player position, competitive level and sex). However, by calculating the number of players with bilateral differences (≥8°) in any hip, knee and ankle ROM measure, the current study found that approximately 20% of futsal players (independent of the player position, competitive level and sex) were identified for HA, HIR, HER, and KF ROMs. There was not a clear pattern in the bilateral differences for the HA, HER, and HIR ROMs so that a similar number of players reported greater values (≥8°) in the dominant and non-dominant limb. Therefore, these bilateral differences may not be attributed to the asymmetrical futsal-specific technical gestures (e.g., kicking and controlling the ball, jumping and turning) that are repeatedly performed during training sessions and matched using mainly the dominant limb. Although a well-founded explanation for this findings has not been found, it might be suggested that these relatively low percentage values of players showing non-patterned bilateral asymmetries (i.e., they are not consistently in favor of the same leg) for the HA, HER, and HIR ROMs may reflect different functional adaptations generated by daily life activities usually performed by players or even an expression of the expected (natural) inter-individual differences attributed to biological (normally distributed) measures (including ROM) as part of being human (Afonso et al., 2020). On the contrary, the bilateral differences for KF ROM reported were mostly in favor of the dominant limb (27 out of 35 cases). A plausible explanation for the KF ROM bilateral differences in favor of the dominant limb identified in this study may be based on the fact that the backswing phase of kicking the ball may reflect in some cases a dynamic stretching for the knee extensor muscles (i.e., quadriceps), which may increase the KF ROM (López-Valenciano et al., 2019). The inter-group analysis revealed that the number of female players showing bilateral differences ≥ 8° in HIR and KF ROMs was approximately double that in males (18% [males] vs. 39% [females] and 18% [males] vs. 33% [females] for HIR and KF ROMs, respectively). Knee ligament injuries often have some long-lasting residual effects and restrictions in HIR and KF ROMs of the injured limb (anecdotical evidence from the authors’ extensive experience in team sports injury prevention and rehabilitation). As a higher incidence of knee ligament injury has been documented in female futsal players then it may be a plausible argument to justify why they presented two-fold more positive cases of significant bilateral differences in the HIR and KF ROMs (Ruiz-Pérez et al., 2021).

Finally, some limitations of this study should be acknowledged. The age distribution of participants was relatively narrow and the goalkeepers’ sample size was small. Moreover, the use of different testing methodologies (i.e., active ROMs) makes comparisons difficult. Likewise, another limitation of this study relies on the fact that only ROM assessments were carried out and hence, it is not possible to determine whether the same bilateral differences could be found in other physical performance tests (e.g., single-leg vertical countermovement jump test and Y-Balance test). The fact that the all the pre-season ROM assessments were not conducted in the same year (due to time and technical constrains) but in four different ones may be also considered a limitation as it cannot be assumed that all pre-seasons presented similar characteristics (e.g., length [weeks], training congestion), which might have had an effect on players neuromuscular properties at the testing timepoint. For females, the phase of their menstrual cycle during testing was not recorded and this may have potentially influenced their ROM scores as fluctuating concentrations of estrogen throughout the menstrual cycle affect musculotendinous stiffness (Eiling et al., 2007; Bell et al., 2009) and joint laxity (Romani et al., 2003). The lower extremities ROM assessment was only carried out at the end of the pre-season phase. Thus, potential changes in hip, knee and ankle ROMs over the course of the in-season phase were not monitored. In this sense, previous study analyzing changes in ADF_*KF*_ and hip (HIR and HER) ROMs over the course of a competitive season for professional male football (Moreno-Pérez et al., 2020) and baseball (Camp et al., 2018; Chan et al., 2020) players, respectively, reported statistically significant (*p* < 0.05) decreases in ADF_*KF*_ and HER ROMs from pre-season to post-season. Although these documented changes in ADF_*KF*_ and HER ROMs were defined as statistically significant, their magnitudes (approximately 3° and 6° for ADF_*KF*_ and HER, respectively) may be considered as not relevant from a clinical standpoint as they did not exceed the value of 1.5 times (80–90% of certainty) the magnitudes of the standard error of the measurement (SEM) reported in previously published reliability studies (Gómez-Jiménez et al., 2015; Gradoz et al., 2018).

## Practical Applications

The findings of this study reveal the existence of large proportions of males (72%) and players from teams engaged in the second division (61%) displaying limited HF_*KE*_ ROMs. Moreover, around 35% of all players showed restricted ADF_*KF*_ ROMs. The results of this study also indicate no significant differences in the ROM for the hip, knee and ankle joints between outfield players and goalkeepers. Likewise, it has been identified a large total number of players with bilateral differences ≥ 8° in HA, HIR, HER and KF ROMs. The number of females with bilateral asymmetries in HIR (18% [males] vs. 39% [females]) and KF (18% [males] vs. 33% [females]) is two time greater than in male players. The potential implications that these restricted HF_*KE*_ and AD_*FKF*_ ROMs and bilateral asymmetries in HA, HIR, HER, and KF ROMs caused by the practice of futsal may have on the physical performance and injury risk of the players warrant future research.

## Data Availability Statement

The raw data supporting the conclusions of this article will be made available by the authors, without undue reservation.

## Ethics Statement

The studies involving human participants were reviewed and approved by Órgano Evaluador de Proyectos, Universidad Miguel Hernández de Elche approved the study (DPS.FAR.02.14). The patients/participants provided their written informed consent to participate in this study.

## Author Contributions

All authors listed have made a substantial, direct and intellectual contribution to the work, and approved it for publication.

## Conflict of Interest

The authors declare that the research was conducted in the absence of any commercial or financial relationships that could be construed as a potential conflict of interest.
